# Data on some socio-economic parameters explaining the movement of extra-EU asylum seekers in Europe

**DOI:** 10.1016/j.dib.2016.11.017

**Published:** 2016-11-09

**Authors:** Silvia Angeloni

**Affiliations:** Department of Economics, University of Molise, Campobasso, Italy

**Keywords:** Asylum seekers, Destination countries, Europe, Regression analysis

## Abstract

This article contains data concerning the movement of extra-EU asylum seekers in Europe. Data used in this paper were collected from the Eurostat database and the UNHCR database. The data consist of some socio-economic features related to 30 European countries where extra-EU asylum seekers have applied for protection. All variables were transformed into their natural logs. The degree of statistical correlation is evaluated from Pearson׳s coefficient, using the 0.05 level of significance. Regression analysis is conducted to identify some socio-economic predictors of countries attracting asylum migration. Six models are presented, where ‘first time asylum applicants’ in 2015 (1,324,215 individuals) in 30 European countries were regressed on 2014 predictors. The multilinear regression model was tested by using data on asylum seekers in 2014, regressed on the same predictors referred to 2013. The data here shared provide a resource for researchers working in the topical field of migration.

**Specifications Table**

**Table:** 

Subject area	*Economics*
More specific subject area	*International Migration, Economic Development, Europe*
Type of data	*Table*
How data was acquired	
Data format	*Raw, analyzed*
Experimental factors	*Several conditions of European destination countries were collected in order to determine their role on attracting extra-EU asylum seekers.*
Experimental features	*The relationship between first asylum applicants and other socio-economic features of European destination countries were determined, after having conducted correlation analysis.*
Data source location	*Luxembourg (Eurostat Database) and Geneve (UNHCR data).*
Data accessibility	*The data are available with this article.*

**Value of the data**

• Some factors behind the trends of asylum claims in destination countries are identified.
• The data can be used by other researchers interested in describing the role played by some conditions as ‘pull factors’ of asylum migration in destination countries.
• The data allow other researchers to extend the statistical analyses by introducing other independent variables.

## 1 Data

For 30 European countries, the following data were retrieved: first asylum applicants; number of refugees per 1000 inhabitants; Gross Domestic Product (GDP) in purchasing power standards (PPS); general government expenditure on social protection (as percentage of GDP); population and its unemployment rate.

Table 1 displays descriptive statistics for the dependent and the independent variables, showing that certain independent variables are strongly correlated to other independent variables.

Table 2 contains six regression models, where ‘first time asylum applicants’ in 2015 (1,324,215 individuals) in 30 European countries were regressed on 2014 predictors.

## 2 Experimental design, materials and methods

Assuming the number of ‘first time asylum applicants’ as dependent variables, some national parameters were hypothesised as independent variables influencing the arrival of asylum seekers.

Data were mainly extracted from the Eurostat database. The information about ‘the number of refugees per 1000 inhabitants’ was retrieved from the UNHCR database.

All variables were transformed into their natural logs [1]. Firstly, correlation coefficients were derived from pairs of variables to describe the strength of associations. The correlation between some independent variables is significant at the 0.01 level. Secondly, the dependent variable was regressed on socio-economic predictors, which was lagged one year to reduce endogeneity concerns [2]. Since all models are in log-log form (with dependent and independent variables both transformed into natural logarithms), the coefficients measure the elasticities of the dependent variable with respect to the predictors [3]. The first five models include only one variable, while the last model includes two independent variables. In particular, the p-value of simple regressions drove the running of multiple regression [4]. Stage by stage, a second predictor was introduced to test whether the regression continues to explain the variable to be predicted beyond information from the preceding stages.

The question of multicollinearity, which can affect multiple regressions, was tested through the variance inflation factor (VIF). There is no multicollinearity problem because the variance inflation factor (VIF) was 1.029, that is less than 10 [1]. The final step in the model-building process was to validate the selected regression model [4]. New data on asylum seekers in 2014 were collected and regressed on the same predictors referred to 2013, always after a logarithmic transformation of independent and dependent variables.

All statistical analyses were further double-checked by using the Statistical Package for Social Science (SPSS) version 18.0.

## Figures and Tables

**Table 1:** Descriptive statistics.

**Variable**	**1**	**2**	**3**	**4**	**5**	**6**
**Log First time asylum seekers - 2015**	1					
**Log Refugees to 1000 inhabitants - 2014**	0.657⁎⁎	1				
**Log GDP (PPS) - 2014**	0.695⁎⁎	0.168	1			
**Log Expenditure on social protection, % GDP - 2014**	0.529⁎⁎	0.351	0.471⁎⁎	1		
**Log Unemployment rate - 2014**	- 0.317	0.480⁎⁎	0.097	0.113	1	
**Log Population** - **2014**	0.600⁎⁎	0.006	0.965⁎⁎	0.370⁎	0.050	1

Note:

⁎ Significant at the 0.05 level (two-tailed);

⁎⁎ Significant at the 0.01 level (two-tailed).

**Table 2:** Regression analyses. Dependent variable: Log First asylum seekers in 2015.

**Variable**	**Model 1**	**Model 2**	**Model 3**	**Model 4**	**Model 5**	**Model 6**
**Log Refugees to 1000 inhabitants**	0.902 (0.196)⁎⁎⁎					0.763 (0.124)⁎⁎⁎
**Log GDP (PPS)**		1.144 (0.223)⁎⁎⁎				0.990 (0.149)⁎⁎⁎
**Log Expenditure on social protection (% GDP)**			5.082 (1.541)⁎⁎			
**Log Population**				1.013 (0.255)⁎⁎⁎		
**Log Unemployment rate**					-1.503 (0.849)⁎	
Adj. *R*^2^	0.411	0.465	0.254	0.337	0.068	0.768
F	21.266⁎⁎⁎	26.197⁎⁎⁎	10.875⁎⁎	15.767⁎⁎⁎	3.126⁎	48.976⁎⁎⁎

Note*:* “*t*” statistic in parentheses.

⁎⁎⁎ *p*<0.01,

⁎⁎ *p*<0.05,

⁎ *p*<0.1.
